# AMPK Regulates Developmental Plasticity through an Endogenous Small RNA Pathway in *Caenorhabditis elegans*

**DOI:** 10.3390/ijms21062238

**Published:** 2020-03-24

**Authors:** Christopher Wong, Richard Roy

**Affiliations:** Department of Biology, McGill University, Montreal, QC H3A 1B1, Canada; christopher.wong8@mail.mcgill.ca

**Keywords:** germ line, quiescence, small RNA, chromatin remodelling, dauer, *C. elegans*

## Abstract

*Caenorhabditis elegans* larvae can undergo developmental arrest upon entry into the dauer stage in response to suboptimal growth conditions. Dauer larvae can exit this stage in replete conditions with no reproductive consequence. During this diapause stage, the metabolic regulator AMP-activated protein kinase (AMPK) ensures that the germ line becomes quiescent to maintain germ cell integrity. Animals that lack all AMPK signalling undergo germline hyperplasia upon entering dauer, while those that recover from this stage become sterile. Neuronal AMPK expression in otherwise AMPK-deficient animals is sufficient for germline quiescence and germ cell integrity and its effects are likely mediated through an endogenous small RNA pathway. Upon impairing small RNA biosynthesis, the post-dauer fertility is restored in AMPK mutants. These data suggest that AMPK may function in neurons to relay a message through small RNAs to the germ cells to alter their quiescence in the dauer stage, thus challenging the permeability of the Weismann barrier.

## 1. Introduction

### 1.1. AMPK and Developmental Plasticity

In *Caenorhabditis elegans* (*C. elegans*), reduced insulin-like signalling induces physiological and morphological changes that can ultimately result in lifespan extension, reproductive delay, and developmental quiescence [1]. The developmental quiescence is triggered at the end of the first larval stage, instructing the animal to execute an alternative third larval stage referred to as “dauer”. The animal will then alter its morphology, behaviour, and metabolism to block all energy-consuming reactions and enhance catabolic processes in order to survive weeks to months without eating. Upon entry into the dauer stage, the animal has no indication of the possible duration of the environmental stress, so every time dauer development is initiated, the changes that occur are essential to protect the animal and preserve the germ line for a period that might last 10–20 times longer than the normal lifespan of the animal.

In a genetic screen conducted to identify factors that are required to maintain germline stem cell quiescence, we found that the *C. elegans* orthologues of AMP-activated protein kinase (AMPK) and liver kinase B1 (LKB1) cooperate to maintain cell cycle quiescence in the germline stem cell population [1]. Mutations in either protein result in an uncontrolled proliferation of germline stem cells during this developmentally-arrested state, while also reducing the duration of survival in the dauer stage [1]. AMPK was identified as an essential factor for the establishment of this quiescence [2]. The loss of any one of the AMPK catalytic α subunits or the β regulatory subunits leads to supernumerary germ cells in the dauer germ line [2]. Quite surprisingly, under optimal growth conditions, mutants that lack all AMPK signalling (*aak(0)*) develop normally; they exhibit normal somatic and germline development similar to wild-type animals and grow into fertile adults. However, if these mutant animals are stressed, they show multiple phenotypes, many of which become apparent during the dauer stage [1,2,3,4].

Although the *aak(0)* mutants do not die immediately as dauer larvae, they are unable to adjust to the energy stress of this diapause stage and this has dramatic effects following recovery. While wild-type animals recover from dauer with little phenotypic consequence, a significant number of AMPK mutants die during dauer exit. Those that survive exhibit highly penetrant sterility and a range of vulval defects [3]. The vulval defects indicate that, in addition to the germ line, the somatic tissues are also sensitive to the loss of AMPK signalling, although the exact mechanism(s) of how AMPK plays such a critical role during dauer is unknown.

### 1.2. C. elegans as a Model Organism to Study AMPK Signalling

In *C. elegans*, AMPK is composed of two catalytic α subunits, *aak-1* and *aak-2*; two β regulatory subunits, *aakb-1* and *aakb-2*; and five γ regulatory subunits called *aakg-1*, *aakg-2*, *aakg-3*, *aakg-4*, and *aakg-5* [5]. At the primary sequence level, these subunits are very similar to their human orthologues. The *C. elegans* and human α subunits share 30% identity and 78% similarity between protein residues [5,6]. The β subunits are also highly conserved at the primary sequence level and share 30% identity and 84% similarity [5,6]. As with most metazoans, AMPK is activated through phosphorylation of its T-loop on Thr243 by PAR-4, the LKB1 orthologue in *C. elegans* [2,4]. Since the subunits of AMPK share a high degree of homology between *C. elegans* and humans, and mutants that completely lack AMPK signalling are viable, our laboratory uses genetic analysis in *C. elegans* to identify novel physiological targets of AMPK by exploring its role in regulating developmental plasticity in response to environmental cues.

In the *C. elegans* hermaphrodite, the larval gonadal primordium consists of four cells: Z1 and Z4 are progenitor cells that will undergo several rounds of mitotic divisions to give rise to all the cells of the somatic gonad. The Z2 and Z3 progenitor cells are referred to as the primordial germ cells and will generate the germline stem cells. These cells will continue to divide throughout the life of the animal to populate the adult bi-lobed gonad with germ cells that will first produce sperm (~300) and then continue to produce oocytes thereafter [5]. Under replete nutrient conditions, animals will develop through the four larval stages L1 through L4, become reproductive adults, and give rise to a full brood (200–300 progeny). These divisions, and the germ line as a whole, are sensitive to environmental cues, such as energetic stressors and/or hormone signalling [7].

During optimal conditions, these cell divisions continue throughout larval development uninterrupted. However, they arrest upon entry into the developmentally-arrested dauer diapause that is specialized for long-term survival and dispersal [8]. This stage is associated with a decreased rate of oxidative metabolism and a modification of lipid and glycogen catabolism [9]. In addition, dauer larvae also have increased transcriptional expression of molecular chaperones and protein components involved in cellular detoxification [10]. These changes in the dauer stage constitute a stress-resistant developmental turnout that is initiated in response to poor environmental/growth conditions, thus providing the larva with an increased chance of long-term survival.

During the dauer stage, the animal is sealed off from its external environment by a highly resistant cuticle, occluding the buccal cavity and making it impossible to feed. Energy is consequently rationed, and metabolism is altered to favour catabolic reactions. AMPK is presumably activated by the reduced food intake typical of the diapause. In response, AMPK activates a number of catabolic enzymes and inhibits anabolic enzymes, such as protein translation and cell proliferation, by inactivating the target of rapamycin (TOR) pathway to reduce energy expenditure [11].

In addition, several cellular processes, such as cell division, and other developmental events are arrested. Germline proliferation is slowed followed by the establishment of a G2 phase cell cycle arrest [2]. The cell cycle quiescence is critical, particularly in the germ line, as cell divisions are energy consuming while replication error frequencies may also be increased due to a limiting, non-sustainable, pool of nucleotides and nucleotide precursors [12,13,14]. Mutations in somatic cells are more easily tolerated in *C. elegans*, but germ cell mutations are problematic as they can be amplified in the germ line and can be inherited throughout subsequent generations.

Both PAR-4 and AMPK are critical to maintain germline quiescence by halting the cell cycle in the germline stem cell population upon entering the dauer stage [2,4], and this process requires both catalytic α subunits of AMPK. There may be some cross talk between the LKB1/AMPK signalling pathway and TOR in several other contexts, but the links between the two signalling pathways are not well defined in *C. elegans*. On the other hand, one of the key effectors of the insulin signalling pathway, the phosphatase and tensin homolog (PTEN) orthologue in *C. elegans* (*daf-18*)*,* can work with AMPK in some contexts, but in the dauer germ line, it appears to function in parallel to AMPK to suppress the germ cell divisions in the dauer stage [2,4].

## 2. Loss of AMPK Results in Germline Defects

### 2.1. Defects in the Dauer Germ Line Result in Post-Dauer Sterility in AMPK Mutants

In AMPK mutant dauer larvae, the integrity of the germ cells is compromised and therefore, they do not produce functional sperm or oocytes, rendering the post-dauer mutants sterile [3]. The morphology and the overall organization of the organ are also aberrant [3]. Under normal circumstances, germ cells will complete meiotic progression to give rise to oocytes with six condensed chromosome bodies (bivalents) that can be visualized by DAPI. However, in the germ line of the post-dauer *aak(0)* mutants, the germ cells enter pachytene but fail to progress to diplotene, where the paired homologous chromosomes normally become apparent [3]. There were no defects in the size of the mitotic zone or the spatiotemporal arrangement of the transition zone [3]. These findings show that the germ cells in post-dauer *aak(0)* mutants fail to exit pachytene and do not undergo diakinesis to produce mature functional gametes. These data indicate that the loss of AMPK during the dauer stage results in a post-dauer meiotic defect that could account for at least some portion of the observed sterility.

Interestingly, reducing the germline hyperplasia in the *aak(0)* dauer larvae is not sufficient to correct the highly penetrant sterility in the post-dauer *aak(0)* adult [3]. By compromising genes that are known to suppress the dauer germline hyperplasia of AMPK mutant animals, including germ line proliferation abnormal (*glp*)*-1,* the resulting reduction of germ cell divisions in the AMPK mutant dauer larvae had little to no effect on the frequency of sterility in the post-dauer animals [3,15]. These findings suggest that the inability to appropriately arrest the germ cell divisions during the dauer stage may not be the cause of the post-dauer sterility typical of the AMPK mutants.

### 2.2. Misregulation of Chromatin Marks and Gene Expression in AMPK Mutants

*C. elegans* larvae that transit through the dauer stage exhibit distinct gene expression profiles that correlate with changes in chromatin marks compared to animals that undergo reproductive development [16,17]. These marks persist in the adult stage and provide a molecular memory of the animal’s life history. These can be represented by a subset of genes that are likely targets of a dauer-dependent reprogramming that may be mediated by a complex RNAi-dependent mechanism [16,17]. In the absence of AMPK signalling, both chromatin remodelling and the activity of endogenous small interfering RNA (endo-siRNA) pathways are misregulated and therefore incorrectly modify gene expression in the dauer and post-dauer animals, resulting in sterility [16,17].

In mammalian cells, AMPK has been shown to regulate gene expression by directly interacting with chromatin and phosphorylating histone H2B in promoter and transcribed regions [18]. Therefore, AMPK activity is essential for adjusting the chromatin landscape to adapt to various cellular stresses. This finding is further corroborated in *C. elegans*, where AMPK signalling also plays a role in modulating the chromatin landscape during periods of stress, namely during the L1 diapause and the dauer arrest [19].

During the dauer stage, AMPK activity is required to ensure that transcriptional activity is modified in accordance with this state of stress in the germ cells, thereby maintaining a state of quiescence during periods when energy must be conserved [3]. In the absence of AMPK signalling, the chromatin landscape and consequent gene expression in the dauer germ line are abnormal [3]. Histone modifications associated with transcription activation (histone 3 lysine 4 trimethylation (H3K4me3) and histone 3 lysine 9 acetylation (H3K9ac)) and repression (histone 3 lysine 9 trimethylation (H3K9me3) and histone 3 lysine 27 trimethylation (H3K27me3)) were all abnormally high [3].

Moreover, the distribution of the chromatin marks in each individual germ cell nucleus was dramatically different in animals that lacked AMPK signalling [3]. Whether this imbalance is a direct cause of the sterility in post-dauer *aak(0)* adults is unknown, but the chromatin marks persist into the post-dauer *aak(0)* adults, where their levels are globally increased compared to the control post-dauer animals [3]. AMPK is thus required by some uncharacterized mechanism to remodel the activating and repressive chromatin marks in the germline chromatin in response to signals specific to the stress associated with the dauer diapause.

The aberrant deposition of the chromatin marks that occurs in the germ cells in the absence of AMPK manifests as changes in gene expression in the dauer larvae and in post-dauer adults [3]. The transcript levels of certain germline-expressed genes (*ppk-2*, *pmk-1*, *spe-26*, *pro-2*, *mek-2*) are significantly altered in the dauer and post-dauer animals compared to those that never transited through the dauer stage [16,17]. Furthermore, similar to the atypical chromatin marks, the abnormal expression of some of the germline-specific genes persists into the post-dauer animals [3]. The misplacement of the chromatin marks in the dauer and post-dauer germ line in the absence of AMPK signalling could cause the germline gene expression to deviate from a gene expression program typical of the dauer stage, resulting in a loss of germline quiescence and germ cell integrity.

## 3. AMPK Regulates Germline Stem Cell Quiescence and Integrity through an Endogenous Small RNA Pathway

Small RNAs control many aspects of gene expression within eukaryotic cells. In *C. elegans*, endo-siRNAs impinge upon specific regions of the chromatin to control which genes will be expressed and which genes will be silenced [20,21,22]. In the last 10 years, a number of Argonaute proteins (RNA binding proteins that are the major effectors downstream of small RNA signals) have been identified that interact with these small RNAs and affect transcription in the nucleus [23,24]. In *C. elegans*, these small RNAs likely respond in an AMPK-dependent manner to adjust the small RNA repertoire in order to remodel the germ line chromatin landscape and couple gene expression to the energetic needs typical of the dauer stage. Not surprisingly, compromise of various components involved in microRNA, germline/nuclear RNAi, or small RNA biogenesis pathways also has consequences on diverse aspects of dauer development, and potentially on the sterility of post-dauer *aak(0)* adults, although not all of them are necessarily associated with AMPK function [3,17].

The compromise of the RNase III-like dicer (DCR-1), its accessory factor RNAi-defective (RDE)-4, and the primary Argonaute protein endogenous-RNAi-deficient Argonaute (ERGO)-1 partially rescues the sterility and vulval defects of the post-dauer *aak(0)* adults and the germline hyperplasia in the *aak(0)* dauer larvae [3]. On the other hand, the compromise of the nuclear Argonaute protein heritable RNA-deficient (HRDE)-1 or the germline licensing Argonaute chromosome segregation and RNAi-deficient (CSR)-1 had no measurable effect on the sterility and germline hyperplasia defect in the *aak(0)* mutants [3].

These findings were surprising considering that the effects of AMPK compromise impact nuclear events, namely the state of the chromatin. Following analysis with genetic mutants and by varying the RNAi strength, it was later determined that the RNAi experiments revealed a specific dosage threshold that resulted in a correction of the sterile phenotype that occurs in the AMPK mutant dauer larvae. If the RNAi was too strong, or alternatively if it was too weak, no effect on the sterility was observed. Moreover, timing of the RNAi application was also important. These findings suggest that the small RNA pathway may be used repeatedly during dauer formation and during recovery. It is critical for the remodelling that must occur first to convert the germ cell chromatin to a quiescent state, and then it may be further required to reactivate germ cell function in response to favourable recovery signals following the diapause [12]. Currently, it is unknown which specific small RNA populations regulate this switch.

Consistent with this, the compromise of *dcr-1* or *rde-4* using RNAi corrects the inappropriate increase and distribution of chromatin marks in the dauer germ line of AMPK mutants [3]. Moreover, the levels of both H3K4me3 and H3K9me3 were significantly reduced in the AMPK mutant dauer larvae with compromised *dcr-1* function [3]. Currently, there is no known direct interaction between AMPK and DCR-1 or RDE-4, although it is noteworthy that the protein sequences of both of these critical RNAi effectors have multiple consensus AMPK phosphorylation motifs. Taken together, these findings suggest that AMPK impinges on the endogenous small RNA pathway to establish germline quiescence and preserve germ cell integrity, by remodelling the chromatin to adjust gene expression in the germ line both during the dauer stage and during recovery to adulthood.

## 4. Somatic Expression of AMPK Rescues the AMPK Mutant Defects

### 4.1. Neurons, Small RNAs, and the Weismann Barrier

Neuronal activity in *C. elegans* can cell nonautonomously regulate a number of developmental and physiological processes, including lifespan and behaviour [25,26]. AMPK was shown to extend lifespan through the neuronal CRTC-1-dependent ((CREB)-regulated transcriptional coactivator-1) remodelling of systemic mitochondrial metabolism [25]. Neuronal CRTC-1/CREB (cAMP response element-binding protein) regulates peripheral metabolism and suppresses the effects of AMPK on systemic mitochondrial metabolism and lifespan extension via a cell nonautonomous catecholamine signal [25]. AMPK phosphorylates CRTC-1 directly to prolong lifespan [25]. When AMPK is unable to phosphorylate CRTC-1 due to the lack of AMPK or mutation of the AMPK phosphorylation motif on CRTC-1, the effects on life span are reversed [25].

Neurons are also at the centre of small RNA-mediated effects that alter behaviour in a transgenerational manner in *C. elegans* [26]. Recent findings have shown that endo-siRNAs can move from the somatic cells into the germ line [26,27]. Endo-siRNAs of neuronal origin can produce a heritable response to regulate behaviour in subsequent generations through the modification of gene expression in the germ line in a manner that is dependent on the activity of the nuclear Argonaute proteins HRDE-1 and CSR-1 [26]. The transmission of these siRNAs is dependent on RDE-4, a neuron-enriched small RNA regulator that was previously shown to play a role in neuronal migration, and learning and memory [28]. The loss of *rde-4* results in a chemotaxis defect, where mutants no longer respond to chemical attractants [28]. The removal of *saeg-2*, which was transgenerationally downregulated in the germline, was shown to rescue the chemotaxis defect of *rde-4* mutants [26]. Thus, neuronal-specific synthesis of RDE-4-dependent endogenous small RNAs affects gene expression cells nonautonomously in affected progeny for up to three generations [26].

### 4.2. Somatic Expression of AMPK is Sufficient to Restore Dauer Germline Quiescence and Post-Dauer Fertility in AMPK Mutants

Recent findings suggest that the soma may regulate changes in the germ line in response to environmental cues through an AMPK-mediated pathway. The ubiquitous expression of one of the two catalytic subunits (*aak-2*) in the soma, restores the fertility of post-dauer AMPK mutants while also rescuing the dauer germline hyperplasia [3]. When AMPK expression was driven by tissue-specific promoters in *aak(0)* mutants, it was found that *aak-2* expression in the neurons and the excretory system was sufficient to rescue the sterility associated with post-dauer animals that lack all AMPK signalling [3]. The identity of the neurons that are capable of mediating this effect has not been determined, nor do we know the precise AMPK targets that are responsible for the correction of the post-dauer sterility. What we can say is that AMPK activity in the soma is sufficient to correct the germline quiescence and germ cell integrity cell nonautonomously during the dauer stage, presumably by correcting the levels and distribution of chromatin marks in AMPK mutant germ cells [3]. These findings challenge the permeability of the Weismann barrier, which states that hereditary information moves only from the germ line to somatic cells, and not vice versa [29].

Other studies have shown that silencing in the germ line is heritable for more than 25 generations after a single parent generation is exposed to neuronal double-stranded RNAs (dsRNAs); this process is highly dependent on the dsRNA-selective importer systemic RNA-deficient (SID)-1 [29,30]. Since SID-1 is involved in the movement of dsRNAs throughout the various cells and tissues, including the neurons, the removal of SID-1 should prevent any misregulated uptake of small RNAs that might have arisen from the neurons into the germ line of AMPK mutant dauer larvae, thereby blocking the establishment of an inappropriate chromatin landscape and consequent errors in gene expression. Consistent with this, the removal of *sid-1* in these AMPK mutants partially restores the fertility in post-dauer adults [3]. *C. elegans* possesses a total of four *sid* family members. The inability to fully restore post-dauer fertility through the removal of *sid-1* suggests that the additional three members of the *sid* gene family could also be involved in the import of small RNAs from the soma into the germ line.

Alternatively, there may also be additional SID-1-independent mechanisms involved in regulating the movement of small RNAs in the AMPK mutants. Studies have shown that the accumulation of the fluorescently-labelled dsRNA resembles the accumulation of yolk proteins (vitellogenins) in proximal oocytes [27]. Yolk proteins are imported through endocytosis and require the protein receptor-mediated endocytosis (RME)-2, a member of the low-density lipoprotein receptor superfamily [30]. When RME-2 function was compromised, import of VIT-2::GFP was blocked along with the fluorescently labelled dsRNAs [27]. Therefore, the import of dsRNAs into oocytes, and subsequently the embryos, at least partially depends on the RME-2-mediated endocytosis machinery required for the import of yolk. This would account for the partial restoration of fertility seen by eliminating *sid-1*, if other import channels that could transport the small RNAs, namely other members of the *rme* gene superfamily, remained intact.

Multiple approaches could be employed to further test the purported permeability of the Weismann barrier in this context. To demonstrate that molecular information generated within the neurons must leave these cells to confer changes in the germ line, the exit of information-containing vesicles could be disrupted genetically by compromising key gene products required for exocytosis. The role of these gene products could then be assessed by quantifying the impact of these genetic changes on the sterility of post-dauer AMPK mutant adults. These same genetic backgrounds could also be used to evaluate how disruption of exocytosis affects the sufficiency of AMPK re-activation in the neurons to correct the germ line integrity of post-dauer AMPK mutants.

Although very informative, these approaches would not provide direct evidence of the movement of information from the soma to the germ line. Molecular labelling of small RNAs in the somatic tissues, such as the neurons or the excretory system, may provide a useful means of following these molecules and identifying their final target tissues. So far, however, this has proven quite challenging due to technical issues with specificity and subsequent tracking. Alternatively, new proximity-labelling techniques might offer a more efficient approach that could facilitate the identification of the molecular nature of this information and the gene products involved in its transmission [31].

## 5. Conclusions

**Figure 1:**
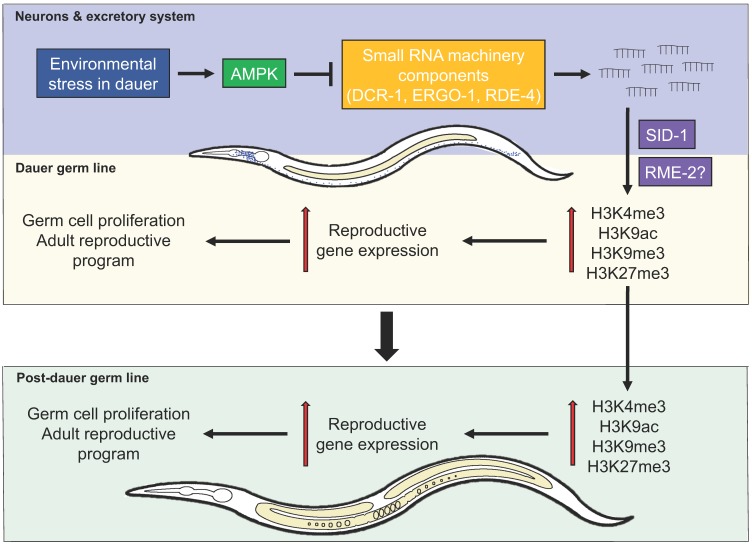
Regulation of germ cell integrity and germline quiescence by the endogenous small RNA pathway in *Caenorhabditis elegans*. Upon encountering environmental stress, the animal will enter the dauer stage. In this stage, AMPK is activated in the somatic tissues to somehow inhibit the biogenesis of endogenous small RNAs, preventing a population of small RNAs from inappropriately specifying chromatin changes typical of reproductive stages during the dauer diapause. These endogenous small RNAs use a double-stranded RNA importer SID-1, and perhaps a vesicle channel protein receptor-mediated endocytosis, RME-2, to import these small RNAs into the germ line. In doing so, AMPK activity promotes the establishment of a chromatin landscape that is consistent with quiescence in the germ line, preserving germ cell integrity over the duration of the dauer stage. When AMPK activity is reduced upon dauer exit, execution of the entire process will ensure the appropriate transition to post-dauer development, with a functional germ line that adequately supports reproduction.

AMPK modulates cellular energy metabolism while transiting through the dauer stage in part by regulating the chromatin landscape and consequent gene expression (Figure 1). In the absence of this master metabolic regulator, animals exhibit germline hyperplasia during the dauer stage while mutants that recover from the diapause show a highly penetrant sterility as well as vulval defects as adults. The distribution of both activating and repressive chromatin marks was upregulated and highly varied in the dauer germ cells. This correlated with a deviation from the normal expression of germline-specific genes in AMPK mutant dauer larvae and this maladaptive expression program persists into the post-dauer animals.

Although we have identified an important link between this protein kinase and the chromatin remodelling that occurs concurrent with dauer entry and exit, the precise targets that are involved and that mediate these changes remain elusive. In addition, the major changes that must occur to the small RNA pathways and the small RNAs themselves must also be under some AMPK control, and this may occur in a collection of neurons that respond to the stress of the dauer stage. Our future work will focus on identifying these key hinge pins, in order to better understand how environmental change can be transduced by AMPK to bring about adaptive, or sometimes even maladaptive, epigenetic responses in the germ line.
